# Formation and thermal stability of two-phase microstructures in Al-containing refractory compositionally complex alloys

**DOI:** 10.1080/14686996.2022.2132118

**Published:** 2022-11-01

**Authors:** Stephan Laube, Alexander Kauffmann, Steven Schellert, Sascha Seils, Aditya Srinivasan Tirunilai, Christian Greiner, Yolita M. Eggeler, Bronislava Gorr, Hans-Juergen Christ, Martin Heilmaier

**Affiliations:** a Institute for Applied Materials (IAM), Karlsruhe Institute of Technology (KIT), Karlsruhe, Germany; b Institut für Werkstofftechnik, Universität Siegen, Siegen, Germany; c Karlsruhe Nano Micro Facility (KNMFi), Karlsruhe Institute of Technology (KIT), Eggenstein-Leopoldshafen, Germany; d MicroTribology Center (µTC), Karlsruhe Institute of Technology (KIT), Karlsruhe, Germany; e Laboratory for Electron Microscopy (LEM), Karlsruhe Institute of Technology (KIT), Karlsruhe, Germany

**Keywords:** Refractory compositionally complex alloys, refractory high entropy alloys, decomposition, separation, nucleation and growth, transmission electron microscopy, atom probe tomography

## Abstract

Phase separation into an A2+B2 two-phase microstructure in refractory compositionally complex alloys (RCCA) has been speculated as being spinodal in nature with continuous chemical distribution during the separation. However, these reactions might instead occur as precipitation by nucleation and growth. In order to unequivocally elucidate the distinct nature of phase separation sequence in RCCA from the system Ta-Mo-Ti-Cr-Al, atom probe tomography and electron microscopy techniques were utilized on samples that were annealed over multiple orders of magnitude in time. The composition 82(TaMoTi)-8Cr-10Al (at.%) was chosen, as it exhibits a two-phase microstructure, with a desired A2 matrix and embedded B2 phase. Quenching the samples from 1200°C resulted in a microstructure consisting of ordered clusters (2 nm) of distinct chemical composition. Subsequent annealing at 800°C to 1000°C leads to an increase in the volume fraction of the precipitating phase, which saturates after 10 h. Further annealing leads to the ripening of the microstructure; however, the absolute size of the precipitates stays <100 nm even after 1000 h. For the investigated conditions, the interface between matrix and precipitate can be considered sharp within the resolution of the applied techniques and no significant change in the transition of chemical composition across the interface is observed. Therefore, the phase separation mechanism is confirmed to be phase nucleation and growth in contrast to the possible spinodal decomposition, as hypothesized for other RCCA systems. The impact of precipitation and coarsening on the hardness of the alloy is discussed.

## GRAPHICAL ABSTRACT

**Figure:**
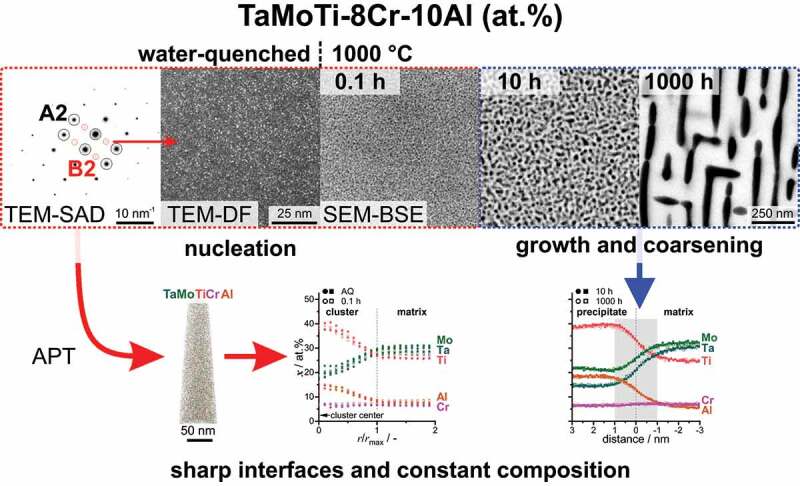

## 1 Introduction

Since the initial publications about high-entropy alloys (HEA), the underlying concepts have gained significant interest due to the possibility of achieving unique combinations of properties over a wide compositional range [1–5]. Research in this field has led to the discovery of alloys with many outstanding properties, such as an exceptional combination of ductility and strength at 4.2 K [6], outstanding yield strength [7]; and corrosion resistance [8,9]; at elevated temperatures. The last two issues were especially interesting in the field of refractory compositionally complex alloys (RCCA). RCCA consist of a combination of mutually soluble refractory metals (possibly in combination with lighter elements [10,11]), which typically results in disordered (*Strukturbericht* A2, W prototype) or ordered (*Strukturbericht* B2, CsCl prototype) body-centered cubic (BCC) crystal structure. Often, significant amounts of lighter elements also lead to the formation of additional phases (e.g. C14, C15, C36, σ or Al_x_Zr_y_) [10,12–15]. Besides alien phases that decorate the grain boundaries, microstructures mimicking that of Ni-based superalloys with matrix channels and blocky precipitates in high-volume fractions can be obtained through appropriate alloying [16]. Some of these alloys can surpass Ni-based superalloys in certain key structural properties, such as high-temperature strength or density [7,10,12]. The B2 phase has been identified as the matrix phase in many of these promising alloys, which explains their lack of ductility at room temperature (RT) [10,17]. This is unfavorable when considering fabrication and practical engineering applications. Apart from the lack of ductility, another key challenge relates phase stability of the precipitating phase as governed by the solvus temperature. To date, this has only been investigated in a few systems [18–24] and found to be lower than the targeted application temperature, implying that microstructural degradation and likely additional coarsening would take place if used at such high temperatures [14,15,25,26].

As an example, the Al-10Mo-Nb-10Ta-TiZr (in at.%) alloy, first introduced by Senkov and co-workers [27,28] in 2014, exhibits an interpenetrating, dual-phase microstructure consisting of a B2 matrix and A2 precipitates [29]. The reported compression yield strength remains high at 1597 MPa and 745 MPa at 800°C and 1000°C, respectively. The density of this alloy is also notably low at 7.4 g/cm^3^ [28], significantly surpassing Ni-based superalloys at around 7.7 to 9.0 g/cm^3^ [30,31]. However, no plasticity is observed at RT. A similar microstructure with cuboidal A2 precipitates within a continuous B2 matrix was also found in 11Al-22Nb-13Ta-28Ti-5V-21Zr [21]. However, investigations of the phase stability by Soni et al. [20] revealed that this microstructure is not stable at 600°C and leads to a phase inversion (matrix phase changes from B2 to A2), which is expected to lead to a dramatic drop in strength. It was assumed that this microstructure develops via spinodal decomposition. The observed phase inversion was speculated to be related to a reduction of the interface energy in combination with changes in elastic stiffness of the B2 phase [20]. The question of whether a spinodal decomposition is responsible for the observed phase separation was recently investigated by Whitfield et al. [32] in the Ti-Nb-Zr subsystem. They concluded that the predicted miscibility gap does not significantly promote phase separation in this specific system. Therefore, the true nature of this phase separation and inversion is not yet fully understood. This issue is not restricted to the Al-Mo-Nb-Ta-Ti-Zr alloy system but extends to other RCCA where a high-temperature single phase transforms into a mixture of disordered and ordered phases.

Alternatively, alloys from the Ta-Mo-Ti-Cr-Al system (with varying Cr and Al concentrations) exhibit a variety of possible microstructures [13,33,34], which results in a high yield strength in excess of 1000 MPa even at 800°C [35]. The equimolar alloy TaMoTiCrAl exhibits a B2 microstructure with thermal anti-phase domain boundaries (APDB) at RT and Laves phase formed at the grain boundaries [34]. This leads to the assumption that the liquid first crystallizes into an A2 structure. At the critical temperature (1109°C [34]), a solid-state ordering transition to B2 occurs by short-range diffusion within the length scale of the unit cells, with thermal APDB formed during this transition. The assumptions were validated by thermodynamic calculations and differential scanning calorimetry (DSC) results in Ref. [34]. Further investigations revealed that the Cr content controls the amount of Cr_2_Ta Laves phase [13], while the Al content controls the ordering to B2 [13,33,35]. However, the complete removal of Cr and Al is presently undesired due to their contributions to the high-temperature corrosion resistance [8,9,36]. Multiple alloys from this system have been investigated so far. The quaternary equimolar MoTiCrAl alloy [34,35] is especially interesting as it exhibits a microstructure similar to the quinary TaMoTiCrAl alloy, although devoid of the detrimental Cr_2_Ta Laves phase. In comparison to the quinary alloy, the quaternary has a lower critical temperature range of application as revealed by thermal analysis. The transition from single-phase A2 to B2 is at 990°C, where the stable crystal structure at RT was observed to be B2 [34]. The Cr-free and Al-lean 83(TaMoTi)-17Al alloy exhibits a multi-phase microstructure, consisting of a B2 matrix with cuboidal A2 precipitates as well as loop-like features (possible segregation to formerly formed APDB) [37]. The impact of Al was further investigated with the 82(TaMoTi)-8Cr-10Al and 77(TaMoTi)-8Cr-15Al alloys [33], which illustrated the idea that by controlling the Al concentration, the alloys can be tailored to exhibit an A2-matrix and B2-precipitate combination or vice versa. 82(TaMoTi)-8Cr-10Al is of special interest because it exhibits a possibly ductile matrix (A2) with strengthening precipitates (B2), mimicking the microstructure of Ni-based superalloys (*Strukturbericht* A1 matrix with L1_2_ precipitates). Despite the promising microstructures of the different Ta-Mo-Ti-Cr-Al alloys, the nature of the phase separation is still not fully understood and the thermodynamic equilibrium composition (and shape) of the phases is unclear, as only results from homogenized and quenched samples of this system were reported until now.

Consequently, the following research questions were assessed in the present study:

1. Does the phase separation subsequent to quenching occur via continuous spinodal decomposition or by discontinuous precipitation associated with nucleation and growth?
2. How do the stages of phase separation influence hardness evolution throughout aging treatment?

Specific focus is on the chemical variation across the interface and the chemical composition of the evolving phases to address the first research question. Tracking the volume fraction and size of the B2 phase allows for an assessment of microstructural changes and their impact on the hardness of the alloy during aging.

## 2 Experimental and materials

The investigated alloy was synthesized by repetitive arc melting inside an AM/0.5 by Edmund Bühler GmbH (Germany) in Ar atmosphere. The pure, bulk elements Ta, Mo, Ti, Cr and Al, with nominal metallic purities of 99.9%, 99.95%, 99.8%, 99.99% and 99.99%, respectively, were provided by chemPUR GmbH (Germany). The cast button was homogenized inside an HTRT 70-600/18 resistance tube furnace by Carbolite Gero GmbH & Co. KG (Germany). The temperature of the heat treatment was 1600°C, while the dwell time was 20 h. An established, continuous Ar flow was maintained throughout the entire heat treatment. Further details regarding the casting and homogenization process are provided elsewhere [33,35]. Small cuboidal samples were cut from the homogenized button via electrical discharge machining. The samples were wrapped in Mo foil and then encapsulated in evacuated fused-silica tubes. The samples inside the fused-silica tubes were annealed inside calibrated L3/S27 resistance furnaces from Nabertherm (Germany). The first heat treatment was performed at 1200°C for 0.5 h, followed by quenching in water. The samples were wrapped and encapsulated again. The second set of heat treatments was conducted for 1 h, 10 h, 100 h or 1000 h at 800°C, 900°C or 1000°C, followed by breaking the ampules submerged in water to quench the samples. A similar set of heat treatments was performed for just 0.1 h by placing the sample at the bottom of a small, cylindrical Al_2_O_3_ crucible to accommodate for the very short heat treatment. To getter O, Ti sponge was placed around and above the samples and the crucible was shut by an Al_2_O_3_ lid. After 0.1 h, the sample and getter material were quenched in water. The getter was not fully oxidized after the heat treatment, indicating that the sample underneath was not oxidized in the process.

In order to achieve a suitable surface for microstructural investigations, samples were first ground using SiC abrasive paper up to grit P4000. Subsequently, metallographic polishing was performed with 3 and 1 µm polycrystalline diamond suspensions for 5 min each. The last preparation step was a mechano-chemical vibratory polishing step for at least 16 h utilizing a non-crystallizing amorphous colloidal silica suspension (with pH of 9.8) provided by Struers GmbH (Germany). Microhardness measurements with an applied load of 0.98 N (HV0.1) were conducted utilizing a Q10A+ Vickers hardness tester from Qness (Austria) and following the recommendations of DIN EN ISO 6507. The indents were placed manually to avoid positions close to grain boundaries and pores. At least 16 indents per sample were evaluated within the supplied software. Electron microscopy was performed using a dual-beam Helios NanoLab™ 650 SEM by FEI (Thermo Fisher Scientific Inc., Oregon, USA), equipped with a secondary electron (SE) and backscattered electron (BSE) detector. The local chemical composition was investigated employing energy-dispersive X-ray spectroscopy (EDS) utilizing an X-Max detector by Oxford Instruments plc (England) at an acceleration voltage of 30 kV. The global chemical composition was investigated by means of inductively coupled plasma optical emission spectrometry (ICP-OES) with an iCAP 7600 DUO device by Thermo Fischer Scientific Inc. The global impurity concentration of N and O was measured through hot gas extraction utilizing a TC500 (LECO Corporation, Michigan, USA). To investigate chemical variations with a near-atomic resolution, three-dimensional atom probe tomography (APT) was employed to determine the chemical identities and the positions of ions. APT samples were manufactured by a state-of-the-art FIB lift-out technique, for further details see Refs. [34,38]. The analysis was performed with a local electrode atom probe LEAP 4000X HR by Cameca SAS (France) in pulsed voltage mode at a sample temperature of 30 K or 50 K. The frequency was set to 125 kHz while the standing voltage was controlled automatically to yield a detection rate of 0.3%. Some of the tips were analyzed by laser measurements utilizing a UV laser (wavelength λ = 355 nm) at a pulse energy of 30 pJ to 50 pJ and a pulse repetition rate of 200 kHz. Within the laser operation mode, the standing voltage is controlled by the software to yield a detection rate of 0.5%. The raw data from the tips was reconstructed, visualized, and further analyzed using the IVAS software (version 3.8.8) by Cameca. For reconstruction, SEM-SE images of the tips were utilized as a reference. In tips from samples annealed for ≥10 h, visualization and analysis of phases were possible by the definition of iso-concentration surfaces at a concentration level of $x_{Al}$ + $x_{Ti}$ = 47 at.%. For shorter annealing durations, a specific cluster analysis following the maximum separation method was necessary. Accumulations with a minimum value $N_{min}$ of Ti and Al ions with a maximum distance $d_{max}$ are regarded as clusters. The aim of this work is to analyze the concentration profile across clusters for which a sufficient number of clusters is necessary. In case of too small values for $d_{max}$, the number of clusters which consist of only a few atoms is very high, which leads to a large scatter in the concentration analysis. If $d_{max}$ is chosen too large, the number of random clusters which are not significant increases drastically. Since the exact size of the clusters was not the focus of this work, $d_{max}$ = 0.35 nm was found to be a compromise. $N_{min}$ was defined individually for each data set by a cluster size analysis, so that no random clusters were included in the analysis. The envelop distance l and the erosion distance $d_{er}$ were set to be equal $d_{max}$. The analyzed amounts of clusters per tip are in the range of 400 to 1400. For a detailed review of the employed methods, the reader is referred to Refs. [39,40]. For the presented proximity histograms [41] and the cluster analysis, the peaks of Ti^2+^ (dominant) and Mo^4+^ were not separated; therefore, a slight over- and underestimation of Ti and Mo, respectively, are present (both within <1 at.%).

The microstructural distribution of both phases was determined within an in-house Matlab code (MathWorks, Inc., Massachusetts, USA). The SEM-BSE micrographs were gently smoothed and the individual histograms equalized. Then, the images were segmented by a threshold evaluation and morphological dilation and erosion. The segmented images were further analyzed to determine the area fraction f as well as the inter-particle spacing of the specific phases. The areal fractions can be regarded as equivalent to volume fractions based on the assumption that the microstructures are isotropic and isometric (all conditions except 900°C/1000 h, 1000°C/100 h, 1000°C/1000 h). The inter-particle spacing was set to be the distance between the geometric centers of each particle subtracted by an area-weighted circle of each particle. In the case of the micrographs from samples that were annealed for 1000 h at 1000°C, the channel width was determined by line-intersection evaluations. The mean precipitate diameter ($d_{mean}$) was calculated by determining the diameter from a circle of area equal to the average precipitate area.

The transmission electron microscopy (TEM) thin foils for post-mortem investigations were prepared by mechanically grinding a disc-shaped sample down to 100 µm thickness utilizing SiC abrasive paper with stepwise finer mesh up to grit P2500. A TenuPol-5 electrolytic polishing device by Struers GmbH was used to thin the samples until sufficient perforation. The TEM investigations were performed with a Talos F200X by Thermo Fisher Scientific Inc. The acceleration voltage was 200 kV and selected area diffraction (SAD), dark-field (DF) and high-angle annular dark-field (HAADF) imaging methods were employed. Further details regarding SEM and TEM procedures are described elsewhere [33,35].

## 3 Results and discussion

### 3.1 Initial microstructure

As reported in Ref. [33], a chemically homogeneous microstructure on the µm-scale with no detectable precipitates can be achieved by a homogenization treatment followed by a 0.5 h heat treatment at 1200°C with subsequent water-quenching. As this is an experimentally reproducible state, it is considered the initial condition for the presented investigations.

**Figure 1.**
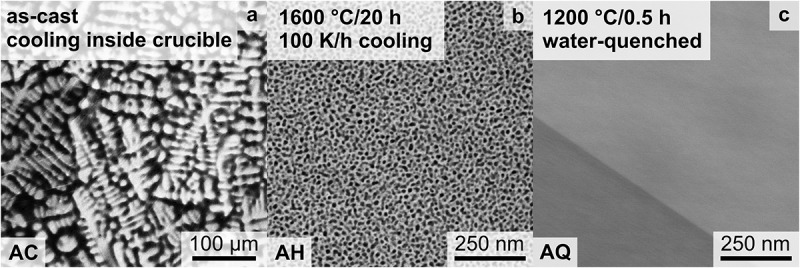
SEM-BSE micrographs of: (a) as-cast (AC); (b) as-homogenized (AH) condition after heat treatment at 1600°C for 20 h with cooling inside the furnace with 100 K/h; (c) as-quenched (AQ); subsequent to the AH condition, the samples were annealed at 1200°C for 0.5 h, followed by quenching in water.

Each of these synthesized/processed states was verified by SEM-BSE imaging (see Figure 1a–c). After homogenization at a slow cooling rate (100 K/h) inside the furnace, the microstructure consists of two distinct phases as depicted in Figure 1b with strong SEM Z contrast. After the additional heat treatment at 1200°C for 0.5 h with subsequent water quenching, precipitates could not be identified, as displayed in Figure 1c, due to insufficient resolution in SEM. Only channeling contrast by different grain orientations is observed. The three displayed sample conditions in Figure 1 will be termed as follows: (a) AC (for as-cast); (b) AH (for as-homogenized); (c) AQ (for as-quenched).

Owing to the importance of chemical composition on phase separation processes, the chemical composition of the AH condition was determined by ICP-OES and is presented in Table 1. Al and Cr are slightly depleted with respect to the desired composition, potentially due to selective evaporation during the arc melting process. Uptake during the melting process as well as annealing at 1600°C in flowing Ar is reasonably low; O and N contents are within the expected range for this synthesis route as reported RCCA are typically in the range of (100 to 500) wt.-ppm [23,33,35,37,42].

**Table 1.** Determined chemical composition ${\overset{ˉ}{x}}_{i}$ of the AH condition by means of inductively coupled plasma-optical emission spectrometry (ICP-OES), presented in at.%. The O and N concentrations by hot gas extraction are given in wt.-ppm.

	${\overset{ˉ}{x}}_{i}$ / at.%					Impurities / wt.-ppm	
Desired composition / at.%	Ta	Mo	Ti	Cr	Al	O	N
27.3Ta-27.3Mo-27.3Ti-8Cr-10Al	28.1	28.3	27.7	7.2	8.7	92 ± 23	45 ± 11

After confirming the chemical starting condition, the AQ condition was further investigated through TEM and APT to elucidate, if water-quenching is sufficient to preserve the high-temperature state (disordered BCC, A2) and results in a supersaturated solid solution, even on the nm-scale.

**Figure 2.**
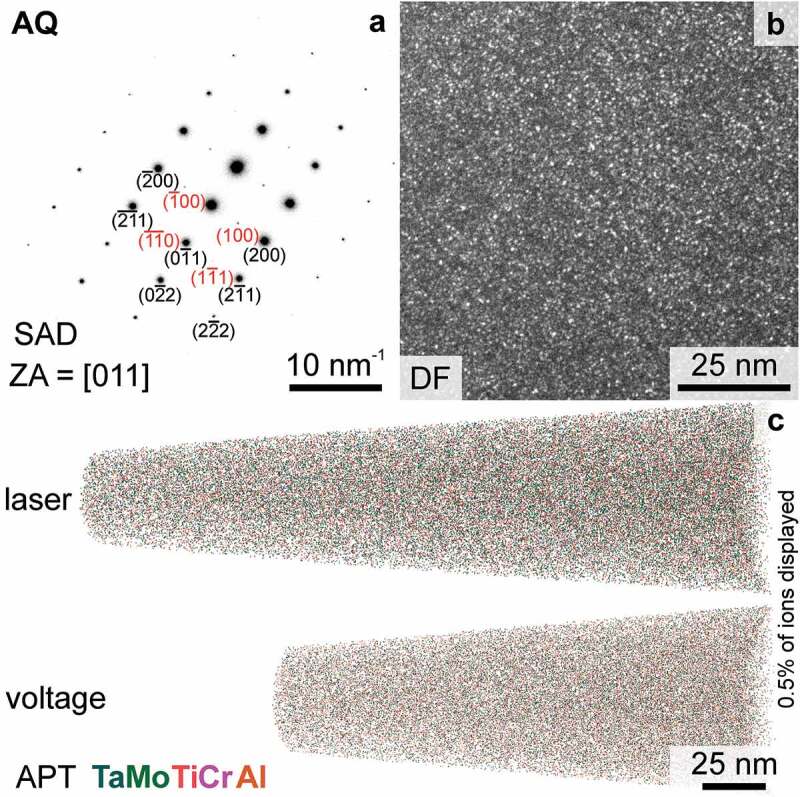
TEM and APT investigations of the AQ condition. (a) TEM-SAD pattern acquired close to the [011] zone axis (ZA). Selected spots are labeled, B2 superlattice spots are labeled in red and A2 fundamental spots are labeled in black. (b) TEM-DF micrograph taken with the objective aperture on a ⟨100⟩ spot (B2 superlattice). (c) Reconstruction presenting the elemental distribution within the examined tips; 0.5% of all detected and assigned ions displayed.

As highlighted in Figure 2a, mild B2 superlattice spots are distinguished in the recorded SAD pattern. Figure 2b depicts a DF micrograph with the objective aperture on a ⟨100⟩ spot (of the B2 superlattice). The bright spots reveal that very small regions in the volume are already ordered in the AQ condition. The size of these spherical features is in the range of 1 nm (utilizing the TEM-DF micrographs). To clarify if the ordered features are linked to a change in chemical composition, APT was performed (spatial and chemical resolution of TEM-based EDS is insufficient in this case). Two 3D reconstructed tips are displayed in Figure 2c. As measurements in voltage mode were not possible in every sample condition in this study, both measurement types will be displayed and examined where possible to highlight the reproducibility of the findings under different experimental conditions. The small difference between the different results of the two APT operation modes illustrates the relevance of the results, even on such length scales. Visually, no segregation or clustering can be discerned for either tip in Figure 2c. Therefore, frequency distribution analysis was performed to clarify, if the ordering is linked to a local ion distribution change.

To quantify the homogeneity of the ion distribution, the Pearson correlation coefficient $\mu_{i}$, for each element i, was calculated within the IVAS software package. The coefficient provides a sample volume-independent quantification of the atomic clustering of solutes in alloys and was first introduced by Moody et al. [43]. The values are defined between 0 and 1, where a complete random solid solution would correspond to $\mu_{i}=0$. The ascertained $\mu_{i}$ values of the AQ condition samples are presented in Table 1.

**Figure 3.**
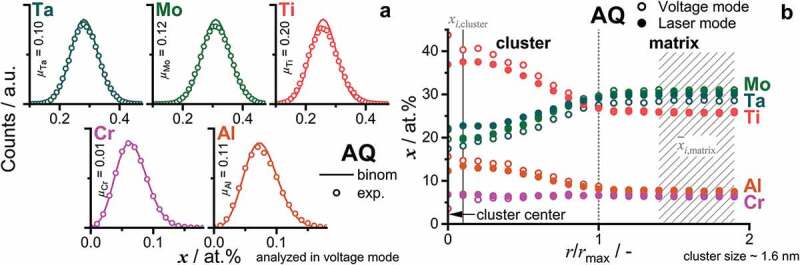
Evaluation of one representative APT tip in the AQ condition. (a) Frequency distribution analysis of each constituent element, the individual $\mu_{i}$ values of which are presented. The binomial distributions are displayed by solid lines, while the experimental distribution is depicted by individual data points (open circles). (b) Cluster concentration profile presented in at.%. The length scale is normalized by the cluster size; therefore, the core of the cluster is at the origin. To exemplify that the difference in APT analysis modes is minor, one data set for laser and one for voltage mode are displayed.

The values for Ta, Mo, Ti and Al are greater than typical values for a chemically homogeneous alloy. As an example, CCA are typically considered homogeneous if $\mu_{i}$ < 0.1 [34,44]. Nevertheless, Cr seems uniformly distributed within all evaluated APT tips. The visualizations of the individual distributions for μ of one tip are displayed in Figure 3a. A broadening of the distributions in all cases except Cr is detected. To further examine the individual distributions, a nearest-neighbor distribution analysis was conducted. The corresponding result indicates that Al and Ti have a slight preference for clustering in Al-Al and Ti-Ti pairs (data not shown here). The remaining ions exhibit a random distance to like ions; nonetheless, the overall tendency is not strong.

Based on the reported composition of the precipitates in the AH condition [33] and the current nearest neighbor distribution analysis, cluster analysis using Al and Ti as core ions was performed to identify a possible phase separation. To estimate the cluster size, the mean value of the extent of the cluster in the three spatial directions is taken. Based on this evaluation, the mean size of the clusters is 1.6 nm, which is in agreement with the TEM observations (Figure 2b). The chemical composition across all determined clusters is presented in Figure 3b. To better account for the different sizes and shapes of the clusters, the length scale is normalized to the approximated size of the clusters. The composition near the core of the clusters and the matrix is presented in Table 2. Ti and Al are enriched, while Ta and Mo are depleted within the clusters. The Cr concentration does not vary, indicating that it does not participate in the initial stage of phase separation. The different analysis modes (laser or voltage) do not have an impact on the concentration profile or absolute concentrations as it is indicated by the minor variation in concentration in Figure 3b. In conclusion, the initiation of phase separation cannot be fully suppressed even for such rapid cooling conditions as performed in this study. This is most likely due to the short diffusion paths that are necessary to form B2 from A2 in the present case; these are only in the order of a few unit cells. Similar ordered clusters were observed in quenched RCCA, which are expected to exhibit a spinodal decomposition, e.g. 8.3Al-16.6Nb-13.3Ta-25Ti-3.3V-16.6Zr [20,24]. However, only a slight change in composition by means of APT was noticed in these alloys. Irrespective of the origin of the ordered clusters, nucleation or spinodal decomposition, they might serve as precursors for the preceding microstructural evolution. This possibility cannot be excluded for the investigated composition, whereas spinodal decomposition is not supported by thermal analysis [33], and it has no consequences on the following investigations.

**Table 2.** Statistical data on ion distribution and chemical composition of clusters. Pearson correlation coefficient $\mu_{i}$ was determined from three APT tips of the AQ condition. Two of the tips were analyzed in laser mode, while one was in voltage mode. Chemical composition $x_{i}$ (in at.%) at the core of the clusters ($x_{i,cluster}$) and matrix (${\overset{ˉ}{x}}_{i,matrix}$), as determined by APT cluster analysis of the tip investigated in voltage mode.

i	Ta	Mo	Ti	Cr	Al
$\mu_{i}$	0.11 ± 0.02	0.15 ± 0.03	0.21 ± 0.01	0.03 ± 0.02	0.13 ± 0.02
${\bar{x}}_{i,matrix}$	28.5 ± 0.1	31.0 ± 0.1	25.9 ± 0.1	6.6 ± 0.1	7.7 ± 0.1
$x_{i,cluster}$	18.1	19.9	40.3	6.9	14.7

### 3.2 Phase separation

**Figure 4.**
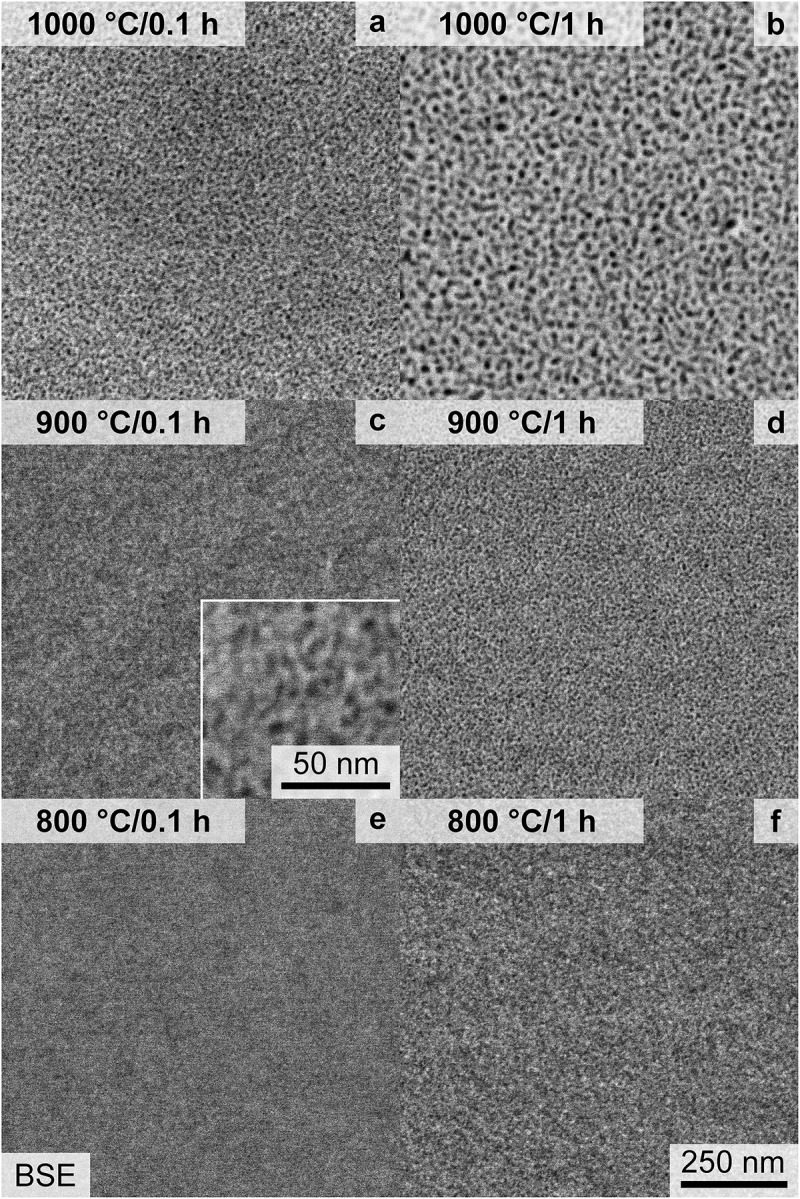
SEM-BSE micrographs with primarily Z contrast taken within a single grain after heat treatment of: (a) 1000°C/0.1 h; (b) 1000°C/1 h; (c) 900°C/0.1 h, with higher magnification inset; (d) 900°C/1 h; (e) 800°C/0.1 h; (f) 800°C/1 h. The same magnification is used for all micrographs, except the inset in (c).

The early stages of phase separation were investigated by means of SEM and APT investigations, on samples that were annealed for 0.1 h and 1 h. Similar studies on other CCA systems analyzed phase separation phenomena up to multiple hours [20,24]; however, it should be noted that in some alloys the spinodal decomposition cannot be suppressed by quenching to RT [45]. The temperatures of 800°C, 900°C and 1000°C were chosen because they are characterized by an unexpected gentle slope in the dH/dT signal in the DSC investigations, as reported in Ref. [33]. At temperatures above 1050°C, it was reported that the crystal structure is single-phase A2; therefore, no higher annealing temperature was chosen. Temperatures below 800°C were not considered in the present study as the phase reactions are practically limited in the present case due to the slow kinetics at these temperatures. For CCA with lower solidus temperatures, like alloys from the Fe-Co-Ni-Mn-Cu [46] or Al-Nb-Ta-Ti-V-Zr [24] systems, even 600°C was enough to observe phase separation phenomena. The homologous temperature, as a rough estimate of diffusivities, is similar among the two mentioned studies at 600°C [24,46] and the 800°C employed in this study on 82(TaMoTi)-8Cr-10Al. The post-annealing micrographs are displayed in Figure 4. After annealing for 0.1 h at 800°C, no distinct phase separation is detected through SEM imaging, as presented by the even Z contrast in Figure 4e. However, annealing at 1000°C and 900°C for 0.1 h results in dark (likely lower Z) and discrete precipitates, as depicted in Figure 4a,c. (for better visibility of the 900°C/0.1 h microstructure, the reader is referred to the higher magnification inset in (c)). The precipitates are evenly distributed within the microstructure and are in the same size range. As depicted in Figure 4b,d,f, the 1 h heat treatment resulted in the development of distinct dark precipitates for all investigated temperatures.

In a previous investigation [33], the question of whether a spinodal decomposition is responsible for the observed multi-phase microstructure was not unequivocally answered since the study relied only on the heat signatures of the phase separation and order processes. The basic concept of spinodal decomposition dates back to the work of Hillert [47], Cahn [48] and Hilliard [49] and is well described in modern textbooks. However, especially in concentrated metallic solutions, the topic is still poorly understood. Therefore, we focused on two distinct features of phase separation processes in our investigations: (i) the sharpness of the boundary as well as (ii) the chemical composition of the individual phases. In the \early stages of separation through spinodal decomposition, the boundary should be broad or blurred dominated by ‘uphill’ diffusion and the deviation from matrix composition should be relatively low. Both features change over time; (i) the boundary gets sharper and (ii) the deviation from matrix composition intensifies. Additionally, during the isothermal treatment, the ordering might only occur, after the composition surpasses the critical point. In contrast, the classical nucleation and growth theory corresponds to (i) a sharp phase boundary throughout the entire process and (ii) a distinct and constant chemical composition of the forming phase right from the nucleation stage and throughout the following growth of the precipitates.

**Figure 5.**
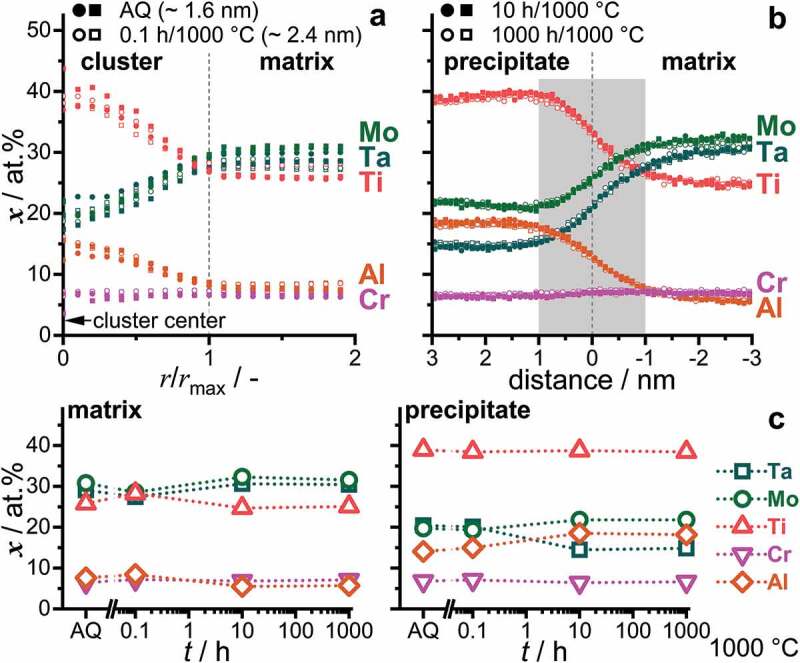
Concentration profiles of the constituent elements over the precipitate/matrix boundary. (a) AQ and 0.1 h, by cluster analysis. AQ data is the same as displayed in Figure 3b. The cluster center is located at *r*/*r*_max_ = 0 and the boundary at one unit of normalized radius. (b) 10 h and 1000 h were evaluated by means of proximity histograms of iso-concentration surfaces with *x*_Ti_ + *x*_Al_ = 47 at.%. (c) Determined concentration in the matrix (left) and precipitates (right) for AQ and 1000°C/0.1 h, 10 h and 1000 h. Error bars smaller than the symbol size are omitted.

Therefore, APT was utilized to achieve a near-atomic resolution. Based on the significant change in the microstructural features observed by SEM imaging, the 1000°C annealed samples were chosen for further investigations by APT. As for the AQ condition, the 1000°C/0.1 h state was analyzed through cluster search and identification. The concentration profiles for both conditions in Figure 5a exhibit a distinct boundary, marked by the change of Ti, Al (increase to the cluster center) and Ta, Mo (depletion to the cluster center), while the Cr concentration does not vary. For longer annealing times (10 h and 1000 h), the precipitates increased in size and iso-concentration surfaces were utilized to distinguish between matrix and precipitates. Proximity histograms (in short ‘proxigrams’ [41]) were evaluated, and the corresponding concentration profiles across the precipitate/matrix interface are depicted in Figure 5b. The concentration profiles for 10 h and 1000 h at 1000°C exhibit virtually the same shape and atomic concentration at a given distance. For both long-term annealed conditions, the boundary has a width of approx. 2 nm.

To correctly assess the boundary profile of the clusters and precipitates, one has to consider the size of the precipitates in comparison to the spatial resolution by APT. As extensively discussed in Ref. [40,50], the spatial resolution of 3D reconstructed APT tips is close to its limits (especially at interfaces) for features less than 2 nm in lateral width. When assuming a sharply developed interface in the 1000°C/1000 h annealed condition independent of the nature of the phase separation process, the 2 nm boundary width can be regarded as a consistent and conservative estimate for the lateral resolution of APT analyses in the present study. Since the thickness of the interface region does not considerably change throughout the investigated stages of phase separation, namely AQ as well as 0.1 h, 10 h and 1000 h, the interface can therefore be regarded as sharp as well as consistent within the resolution of the applied technique throughout the investigated stages of phase separation. In contrast, the experimentally determined interface widths are wider and change upon annealing in the case of spinodal decomposition [51]. Wavelengths/interface widths for CCA are typically in the range of 5 nm to 15 nm at the beginning of decomposition [24,45,52].

To determine the change of composition with annealing time, the evaluated concentrations in the plateau region of the proxigrams and near the centers of the clusters (see Figure 3b) were compiled and displayed in Figure 5c. Only a small change in the composition of the clusters/precipitates between the early stages (AQ and 0.1 h) and the long-term annealed samples is obtained here, in contrast to an intensification of the composition difference to the adjacent regions during spinodal decomposition. The small determined variation can have different, partially superimposed reasons. It might be related to the imprecise chemical resolution for the smallest possible probe size of approx. 2 nm, which leads to a convolution of matrix and precipitate composition. Note that for the initial stages of phase separation (AQ and 0.1 h), the deviation of the chemical composition indeed hints toward such a convolution in the analyses. Furthermore, the potentially broad and temperature-dependent B2 phase field [33] in conjunction with a low diffusivity of Ta might also contribute to slightly changing compositions through the annealing treatment at 1000°C subsequent to the initiation of the phase separation already during quenching. As a comparison for a spinodal CCA, the alloy 15Fe-15Co-20Ni-20Mn-30Cu, investigated by Rao et al. [46], exhibits a significant change in the composition (>20 at.%) of the forming phase with annealing time (2–240 h at 600°C). A similar change in composition during annealing at 600°C was observed for the RCCA 8.3Al-16.6Nb-13.3Ta-25Ti-3.3V-16.6Zr alloy by Dasari and co-workers [24].

Therefore, the preceding analyses lead to the following conclusions: (i) TEM-DF imaging of AQ indicated that the precipitates are already ordered subsequent to quenching. (ii) The interface between the precipitates and the matrix is sharp within the spatial resolution limit of the APT technique for all investigated microstructural conditions. (iii) The change in the composition of the evolving phases is minor for the investigated periods of time (AQ, 0.1 h to 1000 h). Hence, the observed phase separation in 82(TaMoTi)-8Cr-10Al occurs via nucleation and growth as opposed to spinodal decomposition within the investigated temperature range [51].

### 3.3 Growth and coarsening

**Figure 6.**
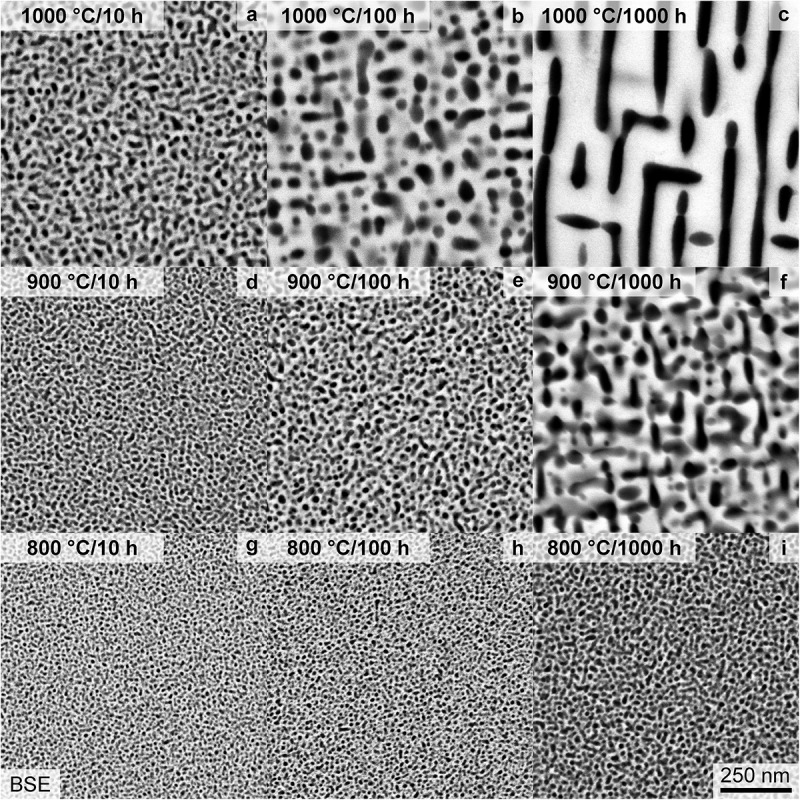
SEM-BSE micrographs with primarily *Z* contrast taken within single grains after a heat treatment of: (a, b, c) 1000°C, (d, e, f) 900°C and (g, h, i) 800°C as well as (a, d, g) 10 h, (b, e, h) 100 h and (c, f, i) 1000 h. The same magnification is used for all micrographs.

To further shed light on the long-term thermal stability of the precipitates, as an important factor for possible high-temperature structural application, the aging characteristics were further analyzed. Samples annealed at 800°C for 10–1000 h exhibit only a slight coarsening of the microstructure, as observed by means of SEM and depicted in Figure 6g–i. Additionally, the precipitates exhibit an almost spherical shape for all investigated conditions. Samples heat-treated at 900°C (displayed in Figure 6d–f) possess a noticeable ripening of the precipitates. After 1000 h, the precipitates undergo an alignment within limited regions, e.g. domains. The 1000°C heat treatment results in coarsening superimposed with a significant change in shape, as depicted in Figure 6a–c. They change from almost spherical (≤10 h, (a)), to cuboidal (100 h, (b)) and finally to raft-like structure (1000 h, (c)) with an alignment confined to domains.

To quantify the coarsening, the SEM-BSE micrographs were binarized, and the relevant parameters, e.g. particle size, inter-particle spacing and area fraction, were evaluated. The area of each precipitate was converted to an equivalent mean diameter $d_{mean}$ for more convenient comparability of particle sizes. In the case of the 1000°C/1000 h condition, a line intercept method perpendicular to the preferred orientation was utilized to determine the inter-particle (channel) spacing.

**Figure 7.**
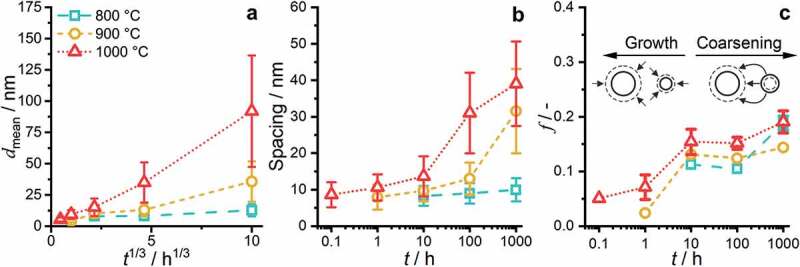
Evaluation of the SEM micrographs based on binarization and morphological operations to separate the two phases. (a) Mean diameter of the particles, $d_{mean}$, as a function of the cube root annealing time, $t^{1/3}$. (b) Inter-particle spacing between the precipitates. (c) Area fraction of the precipitates, f. Schematic differentiation between the growth and coarsening/ripening phase is displayed. The same symbols and colors for each temperature are used throughout this figure.

The precipitate size remains almost constant for annealing at 800°C ($d_{mean}$ ≈ 8 nm to 13 nm). For the higher annealing temperatures, a noticeable increase in the average diameter up to 35 nm and 92 nm for 900°C and 1000°C (both after 1000 h), respectively, was observed. However, it should be noted that the size is still small, especially in comparison to other multi-phase RCCA [19,25,53]. As seen in the compiled $d_{mean}$ in Figure 7a, for a cube root time dependence ($t^{n}$ with n=1/3), an apparent linear trend is observed. Based on log $d_{mean}$ – log t analysis, the time exponents of the 900°C and 1000°C analysis are close to 1/3 with *n* = 0.25 and 0.39, respectively. In the case of the 800°C datasets, the time exponent of 0.1 is significantly smaller, and the double-log linear fit is unreliable.

Regarding the inter-particle spacing, depicted in Figure 7b, the length scale at 800°C does not change significantly from 10 h (8 nm) to 1000 h (10 nm). However, a significant increase is obtained at 900°C, from an average of 13 to 31 nm for 100–1000 h, respectively. A similar trend is described for the spacing at the 1000°C annealing, with a slight increase in interparticle spacing up to 10 h (14 nm), followed by a steeper increase until the 1000 h sample (with 39 nm). The area fraction f of the precipitates seems to be slightly temperature dependent, with higher temperatures leading to higher f in general, as displayed in Figure 7c. The time dependence can be divided into three stages, an increase until 10 h, a plateau between 10 h and 100 h and an increase again until 1000 h.

To rationalize the observed trends, the distinct stages of the precipitation process need to be separated: nucleation, growth and ripening. In the presented cases (t ≥ 0.1 h), the nucleation stage can be regarded as completed, as the AQ condition already exhibits ordered precipitates. Therefore, the growth of the nuclei dominates for the first 10 h in all cases as seen in Figure 7c (for the early 800 h annealing precipitate growth, the reader is referred to an inspection of Figure 4e,f, as no rigorous semiautomatic image evaluation can be performed to these SEM micrographs). After this period, f remains almost constant until 100 h, while the size (e.g. equivalent diameter $d_{mean}$) as well as the inter-particle spacing continuously increase, indicative of dominating ripening/coarsening. The time exponent n≈ 1/3 for the 900°C and 1000°C annealing yields coarsening following an apparent Lifshitz-Slyozov-Wagner (LSW) ripening [54,55]. However, at 800°C, the coarsening does not follow this power-law behavior, even if a nearly constant f is observed between 10 h and 100 h. Conversely, the increase of f from 100 h to 1000 h indicates that the equilibrium volume fraction is not attained after 1000 h and the precipitation reaction is not completed within 1000 h at 800°C. The morphology evolution, especially after 100 h at higher temperatures (Figure 6c,f), is indicative of a relatively minor impact of elastic anisotropy, interfacial energy and lattice misfit. It might be dominated by the increase in the size of the precipitates [56,57]. The plateauing of volume fraction obtained in the experimental data remains unclear and might be due to uncertainties in the segmentation process or a potential transition in the interface condition (partial loss of coherence). Since the diffusion profiles for all elements remain similar throughout the precipitation process, the different diffusivities of the element seem not to be responsible for this plateauing.

**Figure 8.**
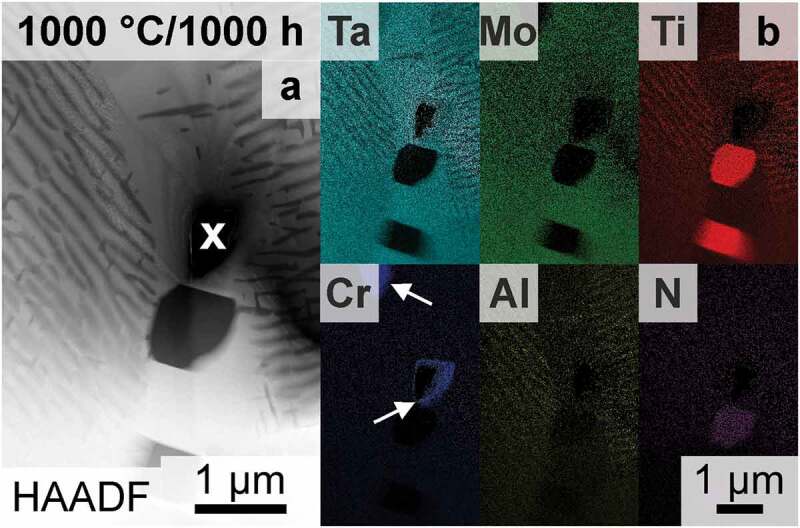
TEM investigations on a selected grain boundary (vertical in the figure) after 1000°C/1000 h; (a) HAADF micrograph of the grain boundary. A hole in the FIB-prepared thin foil is marked with ‘x’. (b) EDS Mappings corresponding to the HAADF micrograph in (a) of the same field of view. The Laves phase is marked with white arrows.

Only the features within the grains have been evaluated and discussed up to this point. However, grain boundaries are often indicators of phase stability, as they enable fast diffusion paths as well as serve heterogeneous nucleation sites. SEM investigations at the grain boundaries of samples annealed at and below 1000°C revealed that two additional phases are present. Therefore, an FIB thin foil of a grain boundary after annealing at 1000°C for 1000 h was examined, and a TEM-HAADF micrograph is shown in Figure 8a. TEM-EDS mappings confirm the presence of Ti- and N-rich regions at the grain boundaries, with an elemental ratio similar to TiN (*Strukturbericht* A1, NaCl prototype) as opposed to Ti_2_N, as depicted in Figure 8b. The observed TiN phase forms preferably at triple junctions or is scattered along grain boundaries. These observations are similar to reports on the AH condition [33]. The global concentration of N is only 45 wt.-ppm (see Table 1) and can be considered quite low, especially compared to the O impurity levels. However, it seems as if N is internally gettered by Ti to form the TiN along the grain boundaries [58,59]. Consequently, the impact on the hardness evaluation can be neglected, as the indents are placed well within the grains [60–62]. The Laves phase (Cr_2_Ta) was detected by means of SEM (not shown here) and TEM (highlighted in Figure 8b by white arrows) in the samples annealed for ≥100 h and ≥900°C at the grain boundaries. However, the volume fraction of the Laves phase is in all cases low (≪ 0.03 vol.%) or even not detected, similar to the investigations in Ref. [59]. Thermodynamic calculations on the (100-*x*)TaMoTiAl-*x*Cr system predict a small amount of Laves phase if the Cr concentration is above 5 at.% (in an otherwise equiatomic alloy) [13], which is in agreement with the experimental results observed here. No secondary phase was detected at the grain boundaries in the AQ condition. However, both phases, TiN and Laves phase, are present in the AH condition [33]. This implies, that the two phases are not stable at temperatures at and above 1200°C as well as exhibit slow formation kinetics [13,60,63]. As showcased for 1000 h (in Figure 8a,b), prolonged annealing (≥100 h) leads to a limited region depleted in Al and Ti close to grain boundaries (e.g. the matrix composition is present). This might be attributed to the formation of the additional phases (which are Ti- and Cr-rich), which change the local composition and therefore the formation of the Ti- and Al-rich B2 phase is not favorable anymore. To summarize, two additional phases form at the grain boundaries though their respective and combined volume fraction is minor for all investigated temperature and time combinations. Their influence on the hardness and/or the bulk precipitation process can be neglected.

### 3.4 Precipitation strengthening

**Figure 9.**
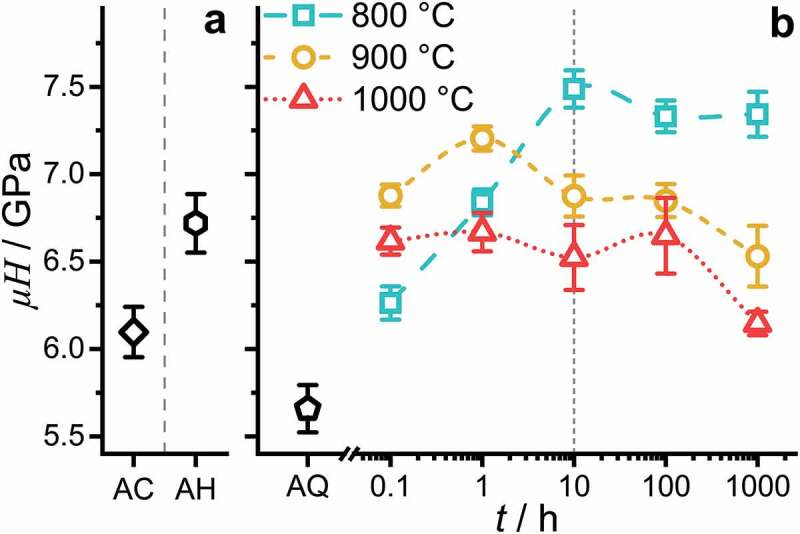
Hardness μH evaluation at RT with a Vickers hardness (HV0.1) testing setup: (a) AC and AH. (b) AQ and after annealing at 800°C, 900°C and 1000°C for 0.1 h, 1 h, 10 h, 100 h and 1000 h followed by quenching in water.

To elucidate the impact of the strengthening by precipitates, hardness μH (with HV0.1 recalculated to GPa) was determined at room temperature. The hardness evaluation is displayed in Figure 9. AC and AH are presented as a reference for the reader (in Figure 9a). As the thermal exposure pathway is uncontrolled for these conditions and restricted to the experimental conditions applied, a further discussion of AC and AH is omitted.

In the AQ condition, a hardness of (5.7 ± 0.1) GPa is obtained. An increase in μH is observed as soon as the samples are annealed. Already after exposure for 0.1 h, the μH increases to 6.3 GPa, 6.9 GPa and 6.6 GPa at temperatures of 800°C, 900°C and 1000°C, respectively. In the case of the 800°C heat treatment, a steady increase of $\mu H^{800{\;}^{\circ }C}$ is observed followed by a hardness plateau at about $\mu H_{plateau}^{800{\;}^{\circ }C}$ ≈ 7.3 GPa. In the case of the 900°C heat treatment, a peak hardness $\mu H_{peak}^{900{\;}^{\circ }C}$ of (7.2 ± 0.1) GPa is reached after 1 h. After the peak, a slight decrease is observed for 10 h and 100 h. After 1000 h, a significant drop to 6.5 GPa occurs. The 1000°C heat treatment resulted in a plateau hardness of $\mu H_{plateau}^{1000{\;}_{\;}^{\circ }C}$ ≈ 6.6 GPa. A decrease to 6.1 GPa is obtained after 1000 h.

The fundamental stages of the precipitation process as revealed in the previous section, namely nucleation, growth and subsequent coarsening, are associated with significant changes in parameters controlling precipitation strengthening: (i) significantly increasing volume fraction in the nucleation and growth stages (see Figure 7b) as well as (ii) significant increase in the particle size and/or inter-particle spacing for the coarsening stage at constant volume fraction (see Figure 7c). Though these two fundamental aspects might be dominating, a quantitative assessment of the precipitation process also requires not yet available parameters like lattice misfit evolution, the evolution of the elastic constants, etc., which might vary during aging. The determination of these parameters requires intensive investigations of the stages presented above and is beyond the scope of the present paper.

Additionally, following other strengthening contributions needs to be discussed in order to deduce the sole impact of precipitates on hardness even for a qualitative assessment [64]: (i) solid solution strengthening, (ii) dislocation strengthening/strengthening by strain hardening, and (iii) yield strength contribution from grain size. As discussed, the composition of the matrix as well as of the precipitates is similar for all stages throughout aging (see Figure 5c). Therefore, a change in substitutional solid solution strengthening can be regarded as negligible. Moreover, the uptake of interstitial atoms (e.g. O, N, …) was verified to be low, and the remaining interstitial N is bonded in the form of TiN close to grain boundaries as shown in the previous section; hence, no significant strengthening contribution is expected in this case. The contribution of dislocation density can be regarded as equally small in all stages of precipitation and coarsening as the microstructures are processed at rather high temperatures. The grain size in all investigated specimens is well above 100 µm (or even up to the mm scale), and all indents were placed manually well inside the grains. Therefore, no contributions from grain boundaries and the additional phases located close to them are expected.

Hence, the only determining change between the samples is the size of the precipitates (by means of $d_{mean}$), shape, inter-particle spacing and area fraction f (equivalent to volume fraction). The precipitates exhibit a B2 crystal structure in all investigated conditions and no spot separation is observed in SAD investigations. Furthermore, no significant change in composition is detected (see Figure 5c). Hence, it can be hypothesized that the interface between precipitates and matrix remains coherent throughout all processing conditions. A superposition from modulus, coherency and order strengthening is then expected [64]. The modulus and coherency strengthening can be regarded as comparable in all stages of precipitation and coarsening, as the chemical compositions remain similar. The anti-phase boundary energy of the B2 phase might be relatively low, as revealed for other multi-phase RCCA [65] (and in contrast to some other binary B2 alloys [66]). In conclusion, resistance against particle cutting might remain small.

Thus, precipitates likely undergo particle cutting at least for the early stages of precipitation (t ≤ 10 h). It remains unclear if the particle-cutting mechanism is dominant also for larger particles or if it changes partially or completely to particle looping [53,67,68]. The plateau hardness between 10 h and 1000 h for 800°C and the slow growth in precipitates’ size at the annealing temperature of 800°C (while maintaining an almost constant f) indicates that the optimal parameter set (for high hardness at RT) under the given circumstances (maximum aging time of 1000 h) is the 800°C/10 h. This is further confirmed by the lower $\mu H_{peak}^{900{\;}^{\circ }C}$ achieved after 1 h, at which the inter-particle spacing is similar to the 800°C/10 h condition but associated with a significantly lower f. It is noteworthy, that the volume fraction of precipitates remains comparably high even for the highest aging temperature (it even slightly increases) indicative of a rather small change in the equilibrium composition of the B2 phase between 800°C and 1000°C. Thus, inter-particle spacing is identified as the determining factor for precipitation strengthening in the present case.

## 4 Conclusions

From the investigations reported in this article, the following conclusions are drawn with respect to the research questions raised:

1. The phase separation in 82(TaMoTi)-8Cr-10Al at temperatures between 800°C and 1000°C subsequent to quenching from 1200°C can be assigned to a diffusion-controlled, discontinuous precipitation process by nucleation and growth with a sharp, moving interface and no chemical modulation of the B2 phase with time.
2. The multiphase microstructure can be considered stable under isothermal conditions for up to 1000 h. Besides the slight coarsening, minor amounts of parasite phases were observed at the grain boundaries. However, *no* catastrophic breakdown of the two-phase microstructure within the grains occurs.
3. The hardness variation as a function of aging temperature and time can be qualitatively explained by factoring in the mean size and volume fraction of the precipitates. Precipitates of $d_{mean}$ ≤ 10 nm and f ≈ 0.1 to 0.2 result in the highest μH at room temperature.
